# How We Treat ANCA-Associated Vasculitis: A Focus on the Maintenance Therapy

**DOI:** 10.3390/jcm14010208

**Published:** 2025-01-02

**Authors:** Dario Roccatello, Roberta Fenoglio, Emanuele De Simone, Savino Sciascia

**Affiliations:** University Center of Excellence on Nephrologic, Rheumatologic and Rare Diseases (ERK-Net, ERN-Reconnet and RITA-ERN Member) with Nephrology and Dialysis Unit and Center of Immuno-Rheumatology and Rare Diseases (CMID), Coordinating Center of the Interregional Network for Rare Diseases of Piedmont and Aosta Valley, San Giovanni Bosco Hub Hospital, ASL Città di Torino and University of Torino, 10154 Turin, Italy; roberta.fenoglio@unito.it (R.F.);

**Keywords:** ANCA vasculitis, relapse, stopping therapy, off therapy, maintenance therapy, rituximab

## Abstract

Recent progress has notably improved outcomes for patients with anti-neutrophil cytoplasmic antibody-associated vasculitis (AAV), namely granulomatosis with polyangiitis and microscopic polyangiitis. Since 2021, several international scientific societies have recommended rituximab (RTX) as the preferred primary treatment for maintaining remission in AAV patients. Decisions regarding retreatment with RTX are based on individual patient risk factors for disease flare-ups and the potential consequences of such flares. In reviewing available evidence and reporting our experiences at G. Bosco Hub Hospital in Turin, Italy, we explore various trials focusing on the maintenance therapy in AAV and discuss areas of unmet need.

## 1. Conventional Approach

Since 2021, several international scientific societies have endorsed rituximab (RTX) as the preferred primary treatment for sustaining remission in patients with ANCA-associated vasculitis (AAV) [1,2]. In the MAINRITSAN1 study, individuals with either initial or recurrent disease were assigned to receive RTX or azathioprine (AZA) following initial treatment with cyclophosphamide [3]. During a 28-month monitoring period, significant relapses were observed in 5% of subjected treated with RTX versus 29% in those receiving AZA with both groups showing comparable safety outcomes. Additionally, in the RITAZAREM trial [4], patients experiencing relapse who were initially treated with RTX either continued with this drug or were switched to AZA for ongoing remission management. The findings indicated a relapse rate of 15% for the RTX group compared to 38% for the AZA group across a follow-up period of at least 36 months with RTX exhibiting fewer severe adverse events.

The mentioned study supported the fact that now RTX is considered the first choice for maintenance therapy in AAV; AZA serves as a viable alternative when RTX is not suitable, and it remains a crucial option for managing AAV during pregnancy due to its safety profile in pregnant women.

At the same time, timing and therapy duration are key factors to considered when planning maintenance therapy in AAV. The REMAIN trial [5] investigated the effects of continuing AZA and glucocorticoids for 48 months versus discontinuing them after 24 months. Results showed that both overall and more severe flares were less frequently observed in those subjects receiving a longer treatment, underscoring the benefits of prolonged therapy with AZA and glucocorticoids for remission maintenance. In the MAINRITSAN2 trial [6], patients were maintained on RTX either at regular six-month intervals or through a tailored approach that adjusted treatment based on ANCA titer changes or the reappearance of CD19+ B lymphocytes. Throughout the follow-up period, the tailored strategy led to fewer infusions: three versus five in the fixed-timing protocol.

Albeit not statistically significant, the patients who received tailored RTX administrations more frequently experienced a relapse—17% versus 10% in the fixed-interval arm.

While the updated international guidelines do not formally endorse this approach [1,2], the use of tailored RTX regimens varies considerably in clinical settings. Furthermore, the SARS-CoV-2 outbreak has significantly altered the use of RTX, prompting physicians to adopt a more flexible approach to B-cell depletion therapy timing. The decision to retreat with B-cell depletion now often considers individual patient risk factors for disease flare-ups and the potential impacts of such flares. This reflects the broader understanding that no single regimen for remission maintenance in AAV fits all patients [7]. A comprehensive literature search was conducted in October 2024 across multiple databases (MEDLINE, OVID) using a structured and inclusive search strategy (keywords: ANCA-associated vasculitis, vasculitis, rituximab, maintenance therapy) to ensure the retrieval of the most up-to-date and relevant references on ANCA-associated vasculitis.

## 2. G. Bosco Hospital Turin Experience

RTX has been effective in inducing remission for both newly diagnosed and relapsing patients, as demonstrated by numerous randomized controlled trials [8,9,10]. Recent studies, both retrospective and prospective, have further explored the efficacy of repeated RTX doses for maintenance therapy [11,12]. Yet, the ideal frequency and dosage of RTX infusions, as well as the optimal duration for maintenance therapy, remain unresolved [13]. It is also uncertain whether RTX dosing should adhere to a fixed schedule or be tailored based on clinical markers such as ANCA titers and B-cell counts or a combination thereof [14]. Moreover, the prolonged effect after the discontinuation of RTX therapy is currently under investigation. In our experience, positive outcomes in patients with refractory AAV treated using an “improved protocol” of RTX involving 4+2 infusions or a combination therapy of RTX with cyclophosphamide (CYC) was observed [15,16,17]. More recently, a case-control study assessed the efficacy and safety of intensified B-cell depletion therapy (IBCDT) versus the traditional CYC-AZA regimen in AAV patients with significant renal impairment (defined as an eGFR of less than 15 mL/min per 1.73 m^2^) [18]. This IBCDT approach, which combined with the “improved protocol” of RTX, CYC, and methylprednisolone pulses, has been successfully employed in treating other severe immune-mediated diseases [18,19,20,21]. In subjects suffering from extremely advanced forms of AAV, one cycle of IBCDT (with no additional therapy as maintenance) proved as effective as the conventional regimen, which includes cyclophosphamide (CYC) for remission induction followed by AZA for maintenance [17]. With a median relapse-free duration of more than 40 months, IBCDT led to the sustained absence of new relapses, which was especially noted in subjects with MPO-AAV. For these patients, adopting a “watchful waiting” approach that includes a strict follow-up of ANCA titers and monitoring B-lymphocytes repopulation is a feasible approach [22].

*How to compare our experience with available evidence?* The MAINRITSAN trial further underscored the effectiveness of RTX over AZA in maintaining remission [3]. This study compared maintenance therapy using either low-dose RTX (administered as two 500 mg doses at 24 weeks, followed by 500 mg every 24 weeks) or AZA after CYC induction. The results highlighted a significant reduction in relapses among patients who received RTX compared to those who were treated with AZA (5% and 29%; *p* < 0.05) over a 28-month follow-up period. Extended observations from the MAINRITSAN trial showed that after 5 years, 57.9% of the RTX-treated patients remained relapse-free versus 37.2% of those treated with AZA (*p* = 0.012) [23]. Subsequent findings from the MAINRITSAN 2 trial, which investigated the ideal frequency for RTX maintenance, revealed that the biomarker-based dosing group, which received RTX less frequently, did not experience a statistically significant difference in relapse rates compared to the fixed-interval dosing group (17.3% vs. 9.9%; *p* = 0.22) [6], suggesting that less frequent dosing might still effectively prevent disease flares.

In a retrospective analysis conducted by the Mayo Clinic, involving 159 patients with MPO-ANCA AAV and renal complications from 1996 to 2015, the study assessed relapse rates, MPO-ANCA status, and the approaches to maintaining remission. It was observed that 46% of patients with a reemergence of MPO-ANCA experienced a relapse with those persistently testing positive for MPO-ANCA showing relapse rates of 39% and 30%. Crucially, patients who consistently tested negative for MPO-ANCA did not experience any relapses, demonstrating that testing negative for MPO-ANCA during the follow-up is associated with up to a total absence of new relapse [24].

Furthermore, in a distinct cohort from North Carolina consisting of 427 patients, 277 individuals (65%) discontinued their treatment with the median time to cessation being 20 months after the initial induction.

Among these, 14% halted their treatment multiple times, and 23% remained off therapy for over five years. Patients who were more inclined to discontinue treatment included those who were MPO-ANCA positive and those diagnosed solely with glomerulonephritis. Interestingly, 194 patients never experienced a relapse, and there were no marked differences in patient characteristics between those who ceased treatment and those who continued [25]. This study further proposed that periods without therapy, when considered as a time-dependent covariate, are linked with roughly half the number of relapses compared to those on continuous therapy, suggesting the potential safety in therapy discontinuation.

**Figure 1:**
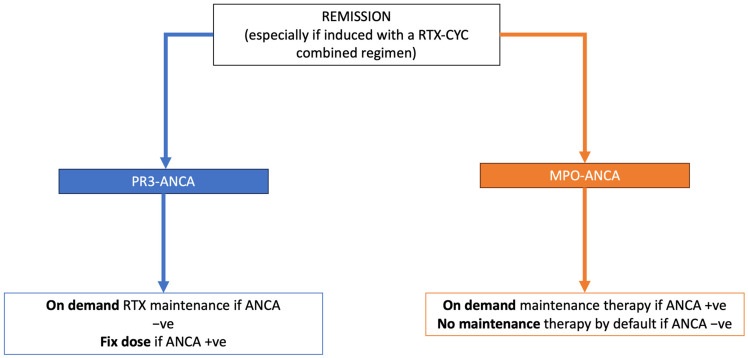
The algorithm proposed by San G. Bosco Hub Hospital in Turin focuses on ANCA specificity and status. In our Turin cohort, a persistently negative ANCA test, particularly with MPO specificity, has demonstrated a strong negative predictive value for relapse. The assessment of histological activity through repeated renal biopsies is considered when there is a re-emergence or exacerbation of urinary abnormalities. Abbreviations: +ve, positive; −ve, negative.

Over the past two decades, the G. Bosco Hospital in Turin has gathered extensive data from a large, prospectively enrolled cohort of 164 patients, of which 127 were recently evaluated. This cohort included 33 patients with granulomatosis with polyangiitis (GPA), all positive for PR3-ANCA, and 94 with microscopic polyangiitis (MPA), of whom 90% were positive for MPO-ANCA. Notably, 30% of these patients had severe renal impairment. Within the MPA-AAV subgroup, 43% experienced no relapses despite not receiving maintenance therapy, underscoring the potential robustness of their initial treatment. IBCDT showed a marked reduction in disease flares compared to other therapeutic regimens. At the point of achieving remission, more than 70% of the subjects tested negative for ANCA compared to the positive patients (17%). Remarkably, the frequency of patients who tested negative and continued to remain negative over time without requiring further maintenance therapy was as high as 50%. This was particularly evident in 56% of the patients with MPO/pANCA vasculitis, and similarly, 41% of those with PR3/cANCA vasculitis demonstrated comparable outcomes. These results underscore the critical role of achieving ANCA negativity following induction therapy [14]. Our approach to maintenance therapy once remission is achieved is schematized in Figure 1. Our findings suggest that for MPO-associated ANCA vasculitis, ongoing maintenance therapy may not be necessary after an effective B-cell depletion induction. The experience at G. Bosco Hospital supports the notion that an intensive protocol for B-cell depletion during remission induction, without routine subsequent immunosuppression, could offer a more favorable approach compared to fixed dosing of RTX, particularly in light of the delayed relapse observed in many cases. This approach could potentially reshape treatment paradigms in managing AAV, especially for certain patient subsets.

## 3. Risk of Relapse

Several clinical factors significantly influence the likelihood of relapse in AAV. Notably, patients at diagnosis who tested positive for antibodies recognizing PR3 are at a heightened risk of experiencing a new recurrence compared to subjects testing positive for MPO [26,27,28,29]. Achieving ANCA negativity after remission induction, irrespective of the initial ANCA subtype, has been strongly associated with a longer relapse-free duration [24,30]. Moreover, the re-appearance of ANCA, along with B-lymphocytes repopulation within a year following the last dose of RTX, are both strong predictors of a potential relapse [10]. Interestingly, subjects with AAV and higher creatinine levels were found to have a lower risk of relapse, suggesting that less severe renal impairment at diagnosis may influence the long-term disease trajectory [31]. These findings underscore the complexity of managing AAV and the importance of tailored therapeutic strategies based on individual risk profiles and disease markers. The impact of a renal flare in patients with initially low eGFR is significantly more severe compared to those with better-preserved kidney function. These patients face an increased risk of progressing to ESKD, as shown by Wester Trejo et al., who demonstrated that subjects with a higher baseline serum creatinine level had a higher risk of ESKD following a relapse compared to those with similar renal impairments who remained in remission [32]. While specific biomarkers for predicting relapse in AAV are still under investigation and not yet routinely used in clinical settings, the presence of active glomerulonephritis during disease relapse has been associated with elevated levels of urinary CD163. Testing for CD163 has shown effectiveness in differentiating between vasculitis-related renal activity and other causes of acute kidney injury [33]. This highlights the ongoing need for precise diagnostic tools to better manage disease progression and tailor treatment approaches, particularly in patients with compromised renal function at diagnosis. In the study of 149 patients with ANCA-associated glomerulonephritis, it was found that hematuria at 6 months is linked with a higher rate of renal recurrence [34]. This suggests that hematuria could serve as a useful indicator of disease activity. However, the relationship between hematuria and disease activity can be complex, as demonstrated by a separate study involving repeat kidney biopsies. In this study, 60% of patients with histologically active disease did not exhibit hematuria, while 59% of those with histologically inactive disease did have hematuria. This indicates that hematuria alone may not reliably predict the histological state of the disease [35]. Additionally, another large study involving more than 500 subjects with renal involvement failed to observe any predictors of kidney recurrence, underscoring the challenges in accurately diagnosing relapses in these conditions. However, maintaining vigilance for changes such as increases in proteinuria and hematuria remains crucial for the early detection of potential relapses [36]. During relapse episodes, erythrocyte sedimentation rate and C-reactive protein are known to increase, particularly during significant disease flares. These markers can therefore be useful in monitoring disease activity. Furthermore, findings from the RAVE trial have added another layer to understanding disease relapse predictors. The trial highlighted that low titers of immuno-regulatory molecules (such as sTim-3, sBTLA and sCD27) were predictive of relapse in subjects with PR3-AAV treated with RTX [37]. This points to the potential utility of these biomarkers in predicting relapse risk, contributing to a more tailored and potentially effective management strategy for patients with this challenging condition.

## 4. Reducing Glucocorticoid Toxicity: The Role of Avacopan

In managing AAV, glucocorticoids (GCs) have historically played a crucial role in controlling disease activity. Recent clinical trials conducted over the past few years have focused on evaluating both new and established therapies with the goal of minimizing cumulative exposure to glucocorticoids [38,39,40]. For example, the PEXIVAS trial [40], a significant study in this area, found that a low-dose GCs protocol was similar in terms of efficacy when compared to standard-GCs protocol in managing the disease. Moreover, this reduced regimen also led to a significant decrease in the total amount of glucocorticoids needed for disease control and notably reduced the risk of serious infections. These findings have had a substantial impact on clinical practice, leading to all three major sets of guidelines—now recommending the lower GCs dosage protocol as the more appropriate approach. This shift is indicative of a broader movement in medicine toward reducing potential side effects associated with long-term glucocorticoid therapy, particularly serious infections, which pose significant risks to patients with compromised immune systems [40]. The ADVOCATE trial marked a significant progression in vasculitis research by implementing avacopan as a replacement for the standard prednisone taper in the treatment of AAV. This trial was particularly notable for its use of a standardized tool, the Glucocorticoid Toxicity Index (GTI), to systematically assess glucocorticoid-related side effects [39,41,42]. Results from the trial demonstrated that patients in the avacopan arm exhibited reduced GTI scores at 3 and 6 months post-treatment initiation, indicating reduced glucocorticoid toxicity [41,42].

Furthermore, avacopan showed superior performance over the standard of care in minimizing glucocorticoid toxicity, achieving significant reductions in GTI scores, including reductions of at least 10 points, which is considered clinically significant [39,41,42]. This suggests that avacopan can be a more effective option in reducing the harmful side effects associated with glucocorticoid use. However, in typical clinical practice, patients often continue receiving high doses of glucocorticoids for extended periods with many remaining on maintenance regimens. Consequently, patients in real-world settings generally are exposed to higher levels of glucocorticoids than those participating in clinical trials. Despite achieving the primary endpoint of discontinuing prednisone by week 21 in the ADVOCATE study, GCs toxicity steadily augmented in both the avacopan and GCs groups with over 90% of participants experiencing significant glucocorticoid toxicity at weeks 13 and 26 [39,43]. These findings underscore the necessity for the ongoing development of treatment strategies that further minimize glucocorticoid exposure while still effectively managing the disease.

## 5. How Can Biomarkers Help Predict Flares?

Predicting disease flares remains a challenge due to their heterogeneous presentation and potential for severe outcomes. As previously mentioned, several studies have provided insights into the utility of biomarkers for identifying patients at risk of relapse, enhancing both monitoring and treatment strategies. It was already stressed how ANCAs are central to the diagnosis and monitoring of AAV. These aspects have been extensively debated [44,45,46]. Among others, according to a study by Han et al. [47], fluctuations in ANCA levels, as measured by PR3- and MPO-ANCA titers, are associated with an increased risk of relapse. However, the predictive value of ANCA levels varies, as not all patients with elevated ANCA titers experience relapses [45]. Single-center cohort studies, such as the one conducted by Kemna and colleagues [46], offer valuable insights into the varying effectiveness of biomarkers across different patient populations. Notably, this study highlights that rising ANCA titers are associated with a greater risk of relapse in patients with renal involvement compared to those without renal disease [46]. This finding highlights the need for combining ANCA trends with clinical parameters to enhance predictive accuracy [48]. A recent meta-analysis emphasized the temporal relationship between rising ANCA titers and relapse risk, noting that flares are more likely to occur within 6 months after a significant rise in ANCA levels. This underscores the importance of longitudinal monitoring of ANCA titers to identify high-risk patients [44].

When referring to potentially novel biomarkers, inflammatory molecules biomarkers such as calprotectin and monocyte chemoattractant protein-1 (MCP-1) have shown promise in predicting disease flares [49,50]. Calprotectin, a marker of neutrophil activation, was identified as a significant predictor of disease activity in a study based on an ad hoc analysis of the MAINRITSAN trial [49]. In detail, an increase in serum calprotectin levels at month 6 compared to baseline during remission-maintenance therapy in AAV was linked to a greater risk of renal function decline within the subsequent 12 months [49].

Urinary MCP-1, a marker of renal inflammation, has also been explored as a non-invasive biomarker for renal vasculitis [50]. Elevated urinary MCP-1 levels were linked to active renal inflammation and predicted renal flares during remission. This organ-specific biomarker offers valuable insights into subclinical disease activity, particularly in patients with renal involvement. In a study by Ohlosson and coworkers [50], they showed that urinary MCP-1 levels were significantly elevated in patients with AAV in the stable phase compared to healthy controls. Among those in remission, patients who experienced subsequent adverse events had notably higher MCP-1 levels than those who did not. While both MCP-1 and IgM levels tended to be higher in patients who relapsed within three months, this observation did not reach statistical significance. Additionally, urinary IL-6 levels were found to correlate with relapse risk, while IL-8 levels were associated with overall disease outcomes. Elevated MCP-1 levels were particularly associated with poor prognosis and a possible tendency for relapse. Notably, the prognostic value of urinary MCP-1 surpassed that of conventional markers such as CRP, BVAS, and ANCA as well as candidate markers like urinary IgM and IL-8. This positions urinary MCP-1 as a promising prognostic biomarker in AAV, warranting further exploration. Recently, a comprehensive protein array analysis of plasma samples from 246 AAV patients in remission identified novel autoantibodies associated with relapse risk [51]. Patients were categorized based on their relapse history, and both descriptive and high-dimensional regression analyses, including LASSO, were applied. Nine autoantibodies were more frequent in patients with multiple relapses compared to those in long-term remission off therapy. Among these, antibodies targeting HFE and SYT5 were consistently associated with relapse across both analytical approaches. These findings highlight HFE and SYT5 autoantibodies as promising candidate biomarkers for predicting relapse in AAV, warranting further validation [51]. A very recent study by Yoon et al. [52] evaluated the role of serum syndecan1 as a biomarker for disease activity and prognosis in patients with AAV. Using a cohort of 79 Korean patients, the study assessed correlations between serum syndecan1 levels at diagnosis and AAV-specific indices, including the BVAS, five-factor score (FFS), 36-item Short-Form Survey (SF-36), and markers of inflammation such as ESR and CRP. Serum syndecan1 levels were significantly associated with higher disease activity (as indicated by BVAS and FFS), poorer functional status (SF-36 PCS and MCS), and elevated acute-phase reactants [52]. Threshold analyses revealed that serum syndecan1 ≥ 76.1 ng/mL was linked to the highest tertile of BVAS, while levels ≥ 60.0 ng/mL predicted upper-half BVAS scores. Notably, patients with serum syndecan1 ≥ 120.1 ng/mL had higher risks of all-cause mortality and lower cumulative survival rates. These findings suggest that serum syndecan1 not only reflects disease activity at diagnosis but also serves as a potential predictor of mortality during follow-up in AAV patients.

At the same time, a better understating of changes and associations with relapse of the circulating autoreactive B cell pool following therapeutic B cell depletion in AAV is providing us with a better understanding of the mechanism related to disease re-activation.

A study by Berti and colleagues [53] investigated the dynamics of the autoreactive B cell pool in PR3-ANCA-positive AAV patients following RTX therapy and its association with relapse risk. The sequential flow cytometry of 148 peripheral blood samples from 23 patients revealed significant changes in B cell composition during recurrence after RTX-induced depletion. At B cell recurrence, PR3+ B cell frequency was significantly higher than baseline, with transitional and naive B cell subsets predominating, while memory subsets were reduced. Notably, patients who relapsed exhibited an enrichment of PR3+ plasmablasts at B cell recurrence, which was associated with shorter relapse-free intervals. Elevated plasmablast frequencies within the PR3+ B cell pool were predictive of relapse within 12 months compared to patients in sustained remission. These findings highlight the role of plasmablast enrichment in the autoreactive B cell pool as a potential biomarker for relapse risk in PR3-ANCA-positive AAV after RTX treatment.

The experiences mentioned above are just a few examples from the growing body of evidence investigating how biomarker monitoring and immunoprofiling could assist physicians in tracking disease activity and ultimately preventing flares.

All in all, given the limitations of individual biomarkers, a composite approach may enhance predictive accuracy. Combining ANCA trends with markers of inflammation (e.g., calprotectin, MCP-1, x syndecan1), circulating autoreactive B cell pool and additional antibodies specificities could provide a more comprehensive assessment of disease activity and relapse risk.

Machine learning models could further optimize biomarker integration by identifying complex patterns in longitudinal data. These tools hold promise for enabling personalized risk stratification and proactive treatment adjustments in AAV management [54].

## 6. Toward Personalized Approaches

In summary, AAV remains a condition with a significant risk of relapse, sometimes occurring years after achieving remission. Historically, the early discontinuation of therapy was associated with increased recurrence frequency and worse prognosis. However, the use of rituximab has notably improved the recurrence rate, shifting perspectives toward the possibility of discontinuing therapy after remission induction—a concept that is increasingly gaining interest among both patients and healthcare providers.

Despite these advancements, the response to cessation of treatment varies widely among patients. Some remain relapse-free for extended periods without ongoing treatment, whereas others experience a reactivation of the immune system, leading to disease flare-ups once immunosuppression is halted. This variability underscores the necessity for a personalized approach to treatment. The effective stratification of patients into low or high-risk categories for relapse is feasible using several factors. These include ANCA status, the degree of circulating B-lymphocytes after therapy, the choice of induction regimen, specific patterns of systems involvement, and emerging biomarkers that signal subclinical activity.

Our ultimate target is tailoring treatment strategies that not only adapt to changing clinical conditions but also reflect the evolving nature of the disease risk based on a combination of intrinsic factors and accumulated clinical experiences. This approach promises a more nuanced and effective management of AAV, potentially reducing unnecessary exposure to long-term immunosuppression while maintaining disease control. After induction therapy, particularly with regimens based on RTX or a combination of RTX and CYC, more than 70% of AAV subjects achieve ANCA negativity. Notably, more than 50% of them, especially those with MPO-ANCA, are able to maintain their negative status without the need for continuous maintenance therapy. Although the rate is somewhat lower in patients with PR3-ANCA, it remains significant. At San G. Bosco Hub Hospital in Turin, the approach includes the vigilant monitoring of ANCA recurrence and CD19+ cell counts (e.g., threshold: >3 CD19 cells) in patients treated with RTX. The repopulation of peripheral B-cells and the associated ANCA titers are pivotal in guiding subsequent management decisions. This strategy underscores the importance of dynamic monitoring to effectively tailor treatment plans based on immunologic markers. Moreover, there is increasing focus on discovering further biomarkers and evaluating the prognostic significance of histological results obtained from serial kidney biopsies. These developments point toward a paradigm shift to more personalized care, which takes into consideration both the risk of relapse and the preferences of the patient in determining the length of maintenance therapy. This individualized approach, however, requires careful implementation. It is crucial to involve patients closely in the treatment process, as they often provide the most timely and accurate detection of early signs of disease reactivation. Engaging patients in their treatment decisions not only enhances adherence but also ensures that therapy is aligned with their unique clinical status and life circumstances, enhancing overall treatment efficacy and patient satisfaction.
